# Whi5 Regulation by Site Specific CDK-Phosphorylation in *Saccharomyces cerevisiae*

**DOI:** 10.1371/journal.pone.0004300

**Published:** 2009-01-28

**Authors:** Michelle V. Wagner, Marcus B. Smolka, Rob A. M. de Bruin, Huilin Zhou, Curt Wittenberg, Steven F. Dowdy

**Affiliations:** 1 Howard Hughes Medical Institute, University of California San Diego School of Medicine, La Jolla, California, United States of America; 2 Department of Cellular & Molecular Medicine, University of California San Diego School of Medicine, La Jolla, California, United States of America; 3 Biomedical Science Graduate Program, University of California San Diego School of Medicine, La Jolla, California, United States of America; 4 Ludwig Institute for Cancer Research, University of California San Diego School of Medicine, La Jolla, California, United States of America; 5 Department of Molecular Biology, The Scripps Research Institute, La Jolla, California, United States of America; 6 Department of Cell Biology, The Scripps Research Institute, La Jolla, California, United States of America; Ordway Research Institute, United States of America

## Abstract

The Whi5 transcriptional repressor is a negative regulator of G1 cell cycle progression in *Saccharomyces cerevisiae* and is functionally equivalent to the Retinoblastoma (Rb) tumor suppressor protein in mammals. In early G1, Whi5 binds to and inhibits SBF (Swi4/Swi6) transcriptional complexes. At Start, Cln:Cdc28 kinases phosphorylate and inactivate Whi5, causing its dissociation from SBF promoters and nuclear export, allowing activation of SBF transcription and entry into late G1. In an analysis of Whi5 phosphorylation, we found that 10 of the 12 putative CDK phosphorylation sites on Whi5 were occupied *in vivo* in asynchronously growing cells. In addition, we identified 6 non-CDK Whi5 phosphorylation sites. Whi5 CDK and non-CDK phosphorylation mutants were functional and able to rescue the small cell size of whi5Δ cells. However, the Whi5 CDK mutant with all 12 putative CDK sites changed to alanine causes a dramatic cell cycle phenotype when expressed with a Swi6 CDK phosphorylation mutant. Mutational analysis of Whi5 determined that only four C-terminal CDK sites were necessary and sufficient for Whi5 inactivation when Swi6 CDK sites were also mutated. Although these four Whi5 CDK sites do not wholly determine Whi5 nuclear export, they do impact regulation of cell size. Taken together, these observations begin to dissect the regulatory role of specific phosphorylation sites on Whi5.

## Introduction

Cell proliferation is a tightly regulated process where extracellular signals and intracellular checkpoints are integrated to control cell growth and division. When conditions for cell division are favorable, cells commit to enter the cell cycle during G1 phase at an irreversible transition termed Start in yeast and the Restriction Point in mammalian cells [1], [2]. In yeast, environmental nutrient availability regulates accumulation of cellular mass to a critical cell size before Start [3]. In mammalian cells, growth factor signaling determines passage through the Restriction Point that is also presumed to be partially responsive to cell size and mass. Failure to properly regulate cell cycle entry can result in abnormal division and lead to development of neoplastic disease [4]. The molecular events determining commitment to cell division in both yeast and mammalian cells involve regulated transcription of groups of genes required for cell cycle progression [5], [6]. In yeast, expression of genes at Start depends on SBF and MBF transcription factor complexes; each consists of the transcriptional coactivator Swi6 and a DNA binding protein, Swi4 or Mbp1, respectively [1]. In general, SBF controls transcription of cell cycle regulatory genes, while genes involved in DNA synthesis and repair are MBF targets [7]. Loss of both Swi4 and Mbp1 results in a permanent G1 arrest and cell lethality, demonstrating the essential function of these transcription factor complexes [8].

In early G1, SBF complexes bind target promoters, but due to binding by the Whi5 transcriptional repressor, do not initiate transcription until Start [9], [10]. The cyclin Cln3 is an activator of cell cycle progression and ensures proper timing of transcription once cells reach a critical cell size [11], [12]. Cells lacking *CLN3* have a large cell size phenotype and are delayed in transcriptional activation and passage through Start [11]–[13]. Whi5 is a key target of Cln3-Cdc28 cyclin dependent kinase (CDK) in transcriptional activation of SBF. Phosphorylation of Whi5 and SBF complexes by Cln3-Cdc28 results in Whi5 dissociation from SBF promoters and nuclear export, allowing for induction of SBF transcription and cell cycle entry [9], [10]. Consequently, cells lacking *WHI5* initiate SBF transcription prematurely, driving cells past Start and resulting in a small cell size [14]. In addition to Whi5, Swi4 and Swi6 are also known targets of CDK phosphorylation and genetic studies implicate Swi6 as a critical target of Cln3 [15]. However, mutation of CDK phosphorylation sites of Swi4 or Swi6 does not interfere with timing of SBF transcription [15]–[17]. Interestingly, a Whi5 CDK phosphorylation mutant causes cell lethality when expressed in a Swi6 CDK phosphorylation mutant background, suggesting that CDK phosphorylation of Whi5 and Swi6 is redundant for transcriptional activation [9]. Swi6 is also subject to inhibitory phosphorylation. During the late G1 to early S-phase transition, Swi6 is phosphorylated by cyclin Clb6-Cdc28 on Ser-160, changing Swi6 cellular localization from nuclear to cytoplasmic [16], [18]. This serves to inactivate SBF transcription outside of G1. It is not currently known what mechanisms repress MBF in early G1 or activate MBF at Start, although the MBF target Nrm1 is responsible for repression of MBF upon exit from G1 [19].

A functionally similar system of transcriptional regulation occurs in mammalian cells. The E2F family of transcription factors control expression of a suite of E2F target genes essential for late G1 cell cycle progression and DNA synthesis [20]. In early G1, E2Fs are bound and repressed by the Retinoblastoma Tumor Suppressor protein (Rb), a functional analogue of Whi5 [21], [22]. At the restriction point, activation of Cyclin E-Cdk2 kinase complexes results in Rb hyper-phosphorylation and inactivation, allowing for cell cycle-dependent E2F transcription [23]. Studies of Rb regulation by phosphorylation show Rb is phosphorylated at low levels, or hypo-phosphorylated, when bound to E2Fs in early G1 [24]. Additionally, it has been proposed by some groups that specific CDK sites on Rb are utilized for inactivation [25], [26], while others suggest a model where a critical threshold of phosphorylation on any of Rb's 16 CDK sites induces inactivation [27], [28]. However, the mechanistic understanding of Rb regulation by phosphorylation remains unclear.

Due to the conserved regulatory pathways controlling G1 transcription between yeast and mammalian cells, investigation of regulation by phosphorylation of Whi5 in yeast could provide insight into mammalian G1 cell cycle and Rb regulation. In an analysis of Whi5 phosphorylation, we determined that Whi5 is also found hypo-phosphorylated in early G1, but that hypo-phosphorylation is not required for Whi5 function. Additionally, we identify four specific CDK sites in Whi5 that are critical for Whi5 inactivation and the regulation of cell size when Swi6 phosphorylation is also prevented. These results demonstrate that regulation of Whi5 by phosphorylation relies on specific CDK sites.

## Materials and Methods

### Yeast Culture and Strains

Cells were grown in standard media containing yeast extract, peptone, and 2% glucose. For induction experiments, cells were grown overnight in media containing 2% raffinose then inoculated into 2% galactose media or galactose plates. A 13x-Myc tag was appended to *WHI5* using the method of Longtine et al [29]. The *WHI5* promoter construct derived from 545 base pairs 5′ of the *WHI5* open reading frame was PCR amplified from genomic DNA and cloned into the pRS413 vector [30].

See Table 1 for a list of yeast strains used in this study.

**Table 1:** 

Yeast Strain	Genotype	Source
MWY01	BY4741 *his3Δ met3Δ ura3Δ leu2Δ*	Brachmann et al.
MWY02	*WHI5*-*13xMyc::kanMX6*	This study
MWY03	*[pRS413 GAL1-WHI5-3xHA]*	This study
MWY04	*[pRS413 GAL1-WHI5*-*12Ala-3xHA]*	This study
MWY05	*[pRS413 GAL1-WHI5*-*13xMyc]*	This study
MWY05	*[pRS413 GAL1-WHI5*-*12Ala-13xMyc]*	This study
MWY06	*[pRS413 GAL1-WHI5*-*6Ala-13xMyc]*	This study
MWY07	*[pRS413 GAL1-WHI5*-*18Ala-13xMyc]*	This study
MWY08	*whi5Δ::kanMX6*	deletion collection
MWY09	*whi5Δ::kanMX6 [pRS413 545pr-WHI5]*	This study
MWY10	*whi5Δ::kanMX6 [pRS413 545pr-WHI5-12Ala]*	This study
MWY11	*whi5Δ::kanMX6 [pRS413 MET3-WHI5]*	This study
MWY12	*whi5Δ::kanMX6 [pRS413 MET3-WHI5-12Ala]*	This study
MWY13	*whi5Δ::kanMX6 [pRS413 MET3-WHI5-6Ala]*	This study
MWY14	*whi5Δ::kanMX6 [pRS413 MET3-WHI5-18Ala]*	This study
MWY15	*[pRS413]*	Brachmann et al.
MWY16	*[pRS413 GAL1-WHI5]*	Costanzo et al.
MWY17	*[pRS413 GAL1-WHI5-12Ala]*	Costanzo et al.
MWY18	*[pRS413 GAL1-WHI5-6Ala]*	This study
MWY19	*[pRS413 GAL1-WHI5-18Ala]*	This study
MWY20	*cln3Δ::kanMX6 [pRS413]*	deletion collection
MWY21	*cln3Δ::kanMX6 [pRS413 GAL1-WHI5]*	This study
MWY22	*cln3Δ::kanMX6 [pRS413 GAL1-WHI5-12Ala]*	This study
MWY23	*cln3Δ::kanMX6 [pRS413 GAL1-WHI5-6Ala]*	This study
MWY24	*cln3Δ::kanMX6 [pRS413 GAL1-WHI5-18Ala]*	This study
MWY25	*swi6Δ::kanMX6 [yEp SWI6-SA4]*	Breeden et al.
MWY26	MWY25 *[pRS413]*	This study
MWY27	MWY25 *[pRS413 GAL1-WHI5]*	This study
MWY28	MWY25 *[pRS413 GAL1-WHI5-12Ala]*	This study
MWY29	MWY25 *[pRS413 GAL1-WHI5-6Ala]*	This study
MWY30	MWY25 *[pRS413 GAL1-WHI5-18Ala]*	This study
MWY31	MWY25 *[pRS413 GAL1-WHI5-7Ala]*	deBruin et al.
MWY32	MWY25 *[pRS413 GAL1-WHI5-4Ala]*	This study
MWY33	MWY25 *[pRS413 GAL1-WHI5-8Ala^1^*]	This study
MWY34	MWY25 *[pRS413 GAL1-WHI5-8Ala^2^]*	This study
MWY35	MWY25 *[pRS413 GAL1-WHI5-1Ala^1^]*	This study
MWY36	MWY25 *[pRS413 GAL1-WHI5-1Ala^2^]*	This study
MWY37	MWY25 *[pRS413 GAL1-WHI5-2Ala^1^]*	This study
MWY38	MWY25 *[pRS413 GAL1-WHI5-2Ala^2^]*	This study
MWY39	MWY25 *[pRS413 GAL1-WHI5-11Ala^1^]*	This study
MWY40	MWY25 *[pRS413 GAL1-WHI5-11Ala^2^]*	This study
MWY41	MWY25 *[pRS413 GAL1-WHI5-10Ala^1^]*	This study
MWY42	MWY25 *[pRS413 GAL1-WHI5-10Ala^2^]*	This study
MWY43	MWY25 *[pRS413 GAL1-WHI5-9Ala^1^]*	This study
MWY44	MWY25 *[pRS413 GAL1-WHI5-9Ala^2^]*	This study
MWY45	MWY25 *[pRS413 GAL1-WHI5-3Ala^1^]*	This study
MWY46	MWY25 *[pRS413 GAL1-WHI5-6Ala^1^]*	This study
MWY47	MWY25 *[pRS413 GAL1-WHI5-3Ala^2^]*	This study
MWY48	MWY25 [pRS413 *GAL1-WHI5*-6Ala^2^]	This study
MWY49	*[pRS413 GAL1-WHI5-GFP]*	This study
MWY50	*[pRS413 GAL1-WHI5-12Ala-GFP]*	This study
MWY51	*[pRS413 GAL1-WHI5-4Ala-GFP]*	This study
MWY52	*[pRS413 GAL1-WHI5-8Ala^1^-GFP]*	This study
MWY53	*msn5Δ::kanMX6 [pRS413 GAL1-WHI5-GFP]*	This study
MWY54	*msn5Δ::kanMX6 [pRS413 GAL1-WHI5-13xMyc]*	This study
MWY55	*swi6Δ::kanMX6 [pRS413 GAL1-WHI5-GFP]*	This study
MWY56	*swi6Δ::kanMX6 [pRS413 GAL1-WHI5-13xMyc]*	This study
MWY57	*GAL1-MSN5 [pRS413 GAL1-WHI5-GFP]*	This study
MWY58	*GAL1-MSN5 [pRS413 GAL1-WHI5-12Ala-GFP]*	This study
MWY59	*swi6Δ::kanMX6*	deletion collection
MWY60	*swi6Δ::kanMX6 whi5Δ::URA3*	This study
MWY61	MWY60 *[yEp SWI6] [pRS413 545pr-WHI5]*	This study
MWY62	MWY60 *[yEp SWI6] [pRS413 545pr-WHI5-12Ala]*	This study
MWY63	MWY60 *[yEp SWI6-SA4] [pRS413 545pr-WHI5]*	This study
MWY64	MWY60 *[yEp SWI6-SA4] [pRS413 545pr-WHI5-12Ala]*	This study
MWY65	MWY60 *[yEp SWI6] [pRS413 545pr-WHI5-4Ala]*	This study
MWY66	MWY60 *[yEp SWI6] [pRS413 545pr-WHI5-8Ala^1^]*	This study
MWY67	MWY60 *[yEp SWI6-SA4] [pRS413 545pr-WHI5-4Ala]*	This study
MWY68	MWY60 *[yEp SWI6-SA4] [pRS413 545pr-WHI5-8Ala^1^]*	This study

### Protein Purification

Cells expressing *GAL1-WHI5-3xHA* or *GAL1-WHI5-12Ala-3xHA* were grown in raffinose then induced with galactose for ten hours. Cells from twelve liters of culture were harvested by centrifugation and drop frozen in liquid nitrogen. Cells were broken with a Waring blender in liquid nitrogen. Resulting powder was resuspended in RIPA buffer with protease and phosphatase inhibitors. Cells debris was pelleted by high-speed centrifugation. Immunoprecipitation with HA affinity resin (Roche) was performed overnight. Immunoprecipitates were washed and boiled in SDS-sample buffer then resuspended in RIPA buffer, diluting the SDS to 1%, before a second immunoprecipitation was performed. Samples from the double immunoprecipitation were separated by 10% SDS-PAGE and stained with Coomassie blue.

### Mass Spectrometry

After in-gel digestion, phosphopeptides were purified by IMAC as previously described [31]. Purified phosphopeptides were analyzed by µLC-ESI-MS/MS on a Thermo Finnigan LTQ quadrupole ion trap mass spectrometer as described [31]. For data analysis, SEQUEST (version 3.4 beta 2) program running on a Sorcerer system (SageN, San Jose, CA) was used for peptide identification. Database search was performed using the budding yeast database. The following variable modifications were considered: +80 Da (phosphorylation) for serine, threonine and tyrosine residues; +16 Da (oxidation) for methionine residues. Up to 4 variable modifications were allowed per peptide and peptide mass tolerance used was 3 Da. A semi-tryptic restriction was applied, and only the top-matched peptides with a probability score above 0.9 were subsequently considered for close inspection. Each MS/MS spectrum that led to phosphopeptide identification was manually verified to confirm all significant ions were accounted for, and then validated.

### Protein Analysis and Phosphatase Treatment

Cells were broken with glass beads in 50 mM Tris, pH 8, 150 mM NaCl, 2 mM EDTA, 10% glycerol, 0.2% NP-40 lysis buffer containing protease (PMSF, aprotinin, leupeptin, benzamidine) and phosphatase inhibitors (Sigma cocktails 1 and 2). Immunoprecipitation was performed with anti-Myc antibody (Santa Cruz) and protein G beads (Zymed) or anti-HA conjugated beads (Roche). After washing with lysis buffer without phosphatase inhibitors, and 50 mM Tris, pH 8, 50 mg/mL BSA, 25 uM DTT buffer, immunoprecipitates were incubated with lambda protein phosphatase (New England Biolabs) in supplied buffer for 1 hour at 30 degrees [32]. Analysis of protein migration utilized 10% SDS-PAGE run for 10–12 cm.

### Cell Size Measurements

Cell size analysis was performed on asynchronous cultures during log-phase growth using a Coulter Z2 Particle Cell Analyzer (Beckman-Coulter). Cultures were briefly sonicated before analysis. Cell size distribution was analyzed with the Z2 AccuComp software (Beckman-Coulter).

### Microscopy

Fluorescence and differential interference contrast microscopy was performed on live cells with a DeltaVision RT Microscope (Applied Precision Life Science) using a 100× oil objective. Image processing was done with ImageJ software and fluorescent and DIC images were overlaid using Adobe Photoshop.

### Cell Synchronization

Cells in log phase growth were washed, resuspended in fresh media containing 5 µg/mL alpha factor (Sigma), and incubated for three hours at 30 degrees. To release, cells were washed twice with cold media then inoculated into warm media. At indicated time points, samples were taken, cooled with ice, sonicated, then analyzed for budding index.

## Results

### Whi5 is phosphorylated on 16 CDK and non-CDK sites *in vivo*

**Figure 1:**
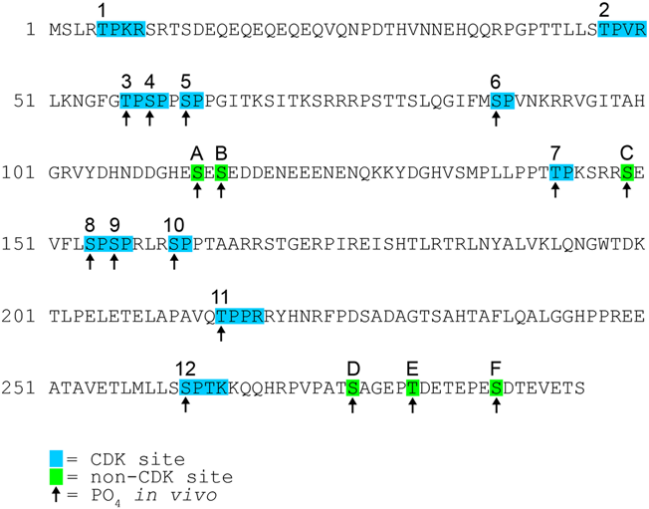
Whi5 is phosphorylated on 16 sites in vivo. The primary amino acid sequence of Whi5 with putative CDK sites boxed in blue. Arrows indicate amino acids found phosphorylated in vivo. Non-CDK sites phosphorylated in vivo are boxed in green.

Whi5 contains 12 putative CDK phosphorylation sites typified by S/T-P(-X-B) and phosphorylation by Cln:Cdk activity has been shown to be important in Whi5 inactivation [9], [10]. de Bruin et al. had previously determined that CDK sites 2, 4, 5, 10 and 12 were phosphorylated *in vivo* from samples of late G1 cells [10]. To identify Whi5 phosphorylation sites *in vivo* in a comprehensive manner, we performed mass spectrometry analysis of Whi5. Wild type Whi5 containing a 3× HA C-terminal tag under the control of the *GAL1* promoter was immunopurified from asynchronously growing cells, digested with trypsin, and phosphopeptides were purified by IMAC resin liquid chromatography enrichment then analyzed by mass spectrometry. Ten of the 12 putative Whi5 CDK phosphorylation sites, numbered 3–12, were identified as being phosphorylated in asynchronous cells (Fig. 1), while phosphorylation of sites 1 and 2 were not detected by our methods. Because de Bruin et al. verified phosphorylation of CDK site 2 [10], and it is possible CDK site 1 is phosphorylated but not detected by our methods, we utilize a Whi5-12Ala phosphorylation mutant in our following studies. Surprisingly, in addition to CDK sites, we also identified 6 other non-CDK serine and threonine residues phosphorylated on Whi5, labeled A to F (Fig. 1). We note that 5 of the 6 non-CDK phosphorylation sites contain an acidic residue distal to the phosphorylated S/T. Mass spectrometry analysis of 3× HA C-terminal tagged mutant Whi5 with all 12 CDK sites changed to alanine (Whi5-12Ala-3xHA) maintained phosphorylation of these 6 non-CDK sites. In total, we identified 16 phosphorylation sites on Whi5 in asynchronously growing cells.

### Whi5 is phosphorylated at all stages of the cell cycle

**Figure 2:**
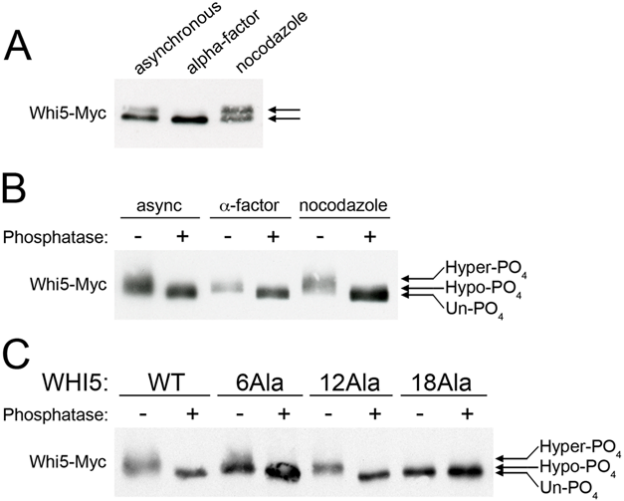
Whi5 is phosphorylated throughout the cell cycle. (A) Cultures of strains expressing Whi5-13xMyc from the endogenous locus were arrested in G1 phase with alpha factor or in metaphase with nocodazole. Western blot of Whi5-13xMyc shows two different migrating forms, indicated by arrows. (B) Western blot of immunopurified Whi5-13xMyc treated with or without lambda phosphatase. Three different migrating forms indicated by arrows represent unphosphorylated, hypo-phosphorylated, and hyper-phosphorylated Whi5. (C) Strains carrying CEN plasmids of phosphorylation mutants of Whi5-13xMyc under the *GAL1* promoter were grown in galatcose before immunopurification. Whi5-WT, non CDK mutant 6Ala, CDK mutant 12Ala, and CDK/non-CDK mutant 18Ala were treated with phosphatase as in B. Arrows indicate migration of three different phosphorylation forms.

We next analyzed the cell cycle dependency of Whi5 phosphorylation, utilizing a strain containing a C-terminal 3x-Myc tag integrated at the *WHI5* locus. In lysates from asynchronously growing cells, Whi5-13xMyc protein migrates as a doublet (Fig. 2A). However, in cells arrested in G1 phase by mating pheromone, where CDK activity is inhibited by Far1 [33], [34], Whi5-13xMyc migrates as a single, faster migrating band. In contrast, in cells arrested in M phase by nocodazole, where CDK activity is high, the slower migrating species was enriched. To test whether the difference in migration of the Whi5 bands was due to phosphorylation, immunoprecipitates of Whi5-13xMyc were treated with lambda phosphatase (Fig. 2B). Upon phosphatase treatment, both the slower and faster migrating species of Whi5 from asynchronous cells collapsed into a third, fastest migrating un-phosphorylated band. Phosphatase treatment of Whi5-13xMyc from mating pheromone and nocodazole-arrested cells both induced appearance of the un-phosphorylated band. Thus, Whi5 can be separated into three species: the slowest migrating form of Whi5, designated hyper-phosphorylated; the middle form, designated hypo-phosphorylated; and the fastest form, designated un-phosphorylated. We note that the un-phosphorylated form of Whi5 was not found at any cell cycle stage or even in mating pheromone arrested cells. In addition, we observe that Whi5 from mating pheromone arrested cells with inhibited CDK activity shifted downward after phosphatase treatment, suggesting the presence of non-CDK phosphorylation events.

The phosphorylation status of Whi5 mutants was also analyzed by phosphatase treatment. Wild type Whi5 or phosphorylation mutants containing a C-terminal 13xMyc tag were expressed from the GAL1 promoter on CEN plasmids. In samples of asynchronous cells, mutation of the 6 non-CDK sites (A–F) to alanine did not effect the migration of Whi5 compared to wild type Whi5 (Fig. 2C). Consistent with the mass spec analysis, mutation of Whi5 CDK sites 1–12 to alanine resulted in a single hypo-phosphorylated band that increased mobility after phosphatase treatment. Since both Whi5-6Ala and Whi5-12Ala were found phosphorylated, the non-CDK sites A–F are not essential for CDK site phosphorylation, and *vice versa*. Finally, when all 12 CDK and 6 non-CDK sites were mutated to alanine in Whi5-18Ala, migration did not change upon phosphatase treatment. In addition, *in vivo* orthophosphate labeling verified that both Whi5-6A and Whi5-12Ala are phosphorylated, although to a lesser extent than wild type Whi5, while phosphorylation of Whi5-18Ala was negligible (data not shown).

### Functional Analysis of Whi5 phosphorylation mutants

**Figure 3:**
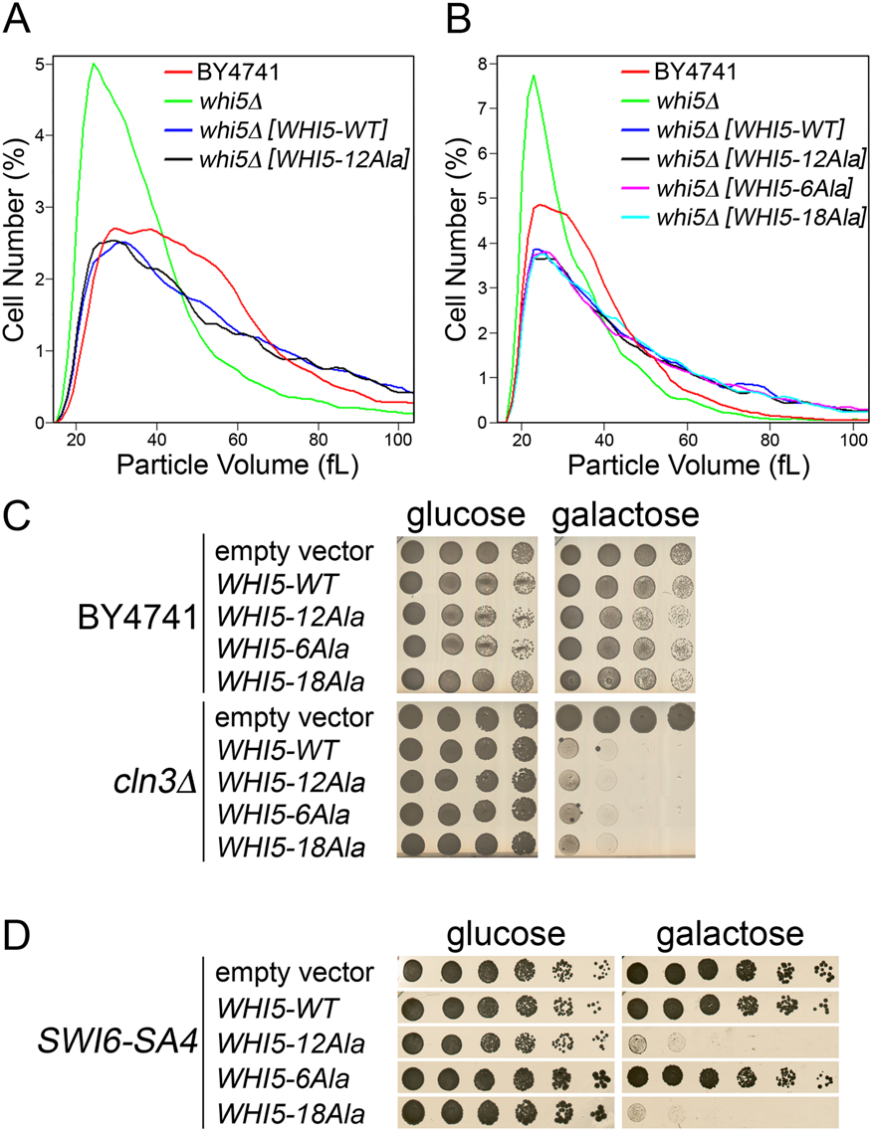
Whi5 hypo-phosphorylation is not necessary for function. (A) Size analysis of *whi5Δ* cells with CEN plasmids expressing Whi5-WT or Whi5-12Ala (CDK mutant) from the *WHI5* promoter, a 545 bp fragment from sequence directly 5′ of the *WHI5* open reading frame. (B) Size analysis of *whi5Δ* cells expressing Whi5-WT, Whi5-12Ala (CDK mutant), Whi5-6Ala (non CDK mutant), or Whi5-18A (CDK and non-CDK mutant) from the *MET3* promoter on a CEN plasmid. (C) Wild type cells or *cln3Δ* cells with empty vector or CEN plasmid constructs of Whi5-WT, Whi5-12Ala, Whi5-6Ala, or Whi5-18Ala under control of the *GAL1* promoter. Cells were spotted in serial five fold dilutions on glucose or galactose media and incubated 48 hours. (D) *swi6Δ [SWI6-SA4]* cells bearing either empty vector or CEN plasmid constructs of Whi5-WT, Whi5-12Ala, Whi5-6Ala, or Whi5-18Ala under the *GAL1* promoter. Cells were spotted in serial five fold dilutions on glucose or galactose media and incubated 48 hours.

*whi5Δ* cells exhibit a small cell size phenotype [14]. To assess the functionality of Whi5 phosphorylation mutants, we assayed for their ability to rescue *whi5Δ* small size (Fig. 3A). Wild type parent strain BY4741 had a mean cell size distribution of 48.2 fL, while the mean size of *whi5Δ* cells was 36.2 fL in this experiment. Introduction of wild type *WHI5* on a CEN plasmid under its native promoter into *whi5Δ* cells rescues the small cell size defect and returned the culture to a mean cell size of 53.2 fL. Expression of *WHI5-12Ala* also rescued the small cell size phenotype, with a mean cell size of 53.3 fL. Importantly, expression of *WHI5-12Ala* from its own promoter did not result in a larger cell size than *WHI5-WT*. This suggests CDK phosphorylation of Whi5 is not essential for SBF activation or timing of Start, as loss of Whi5 CDK phosphorylation does not delay cell cycle progression. Mutants in the non-CDK phosphorylation sites of Whi5 were also assayed for ability to rescue *whi5Δ* cell size. In this experiment, where cells are grown in synthetic media, parent strain BY4741 had a mean cell size of 35.3 fL, while *whi5Δ* cells were 31.5 fL. Expression of *WHI5* wild type, *WHI5-12Ala* (CDK), *WHI5-6Ala* (non-CDK), or *WHI5-18Ala* (CDK and non-CDK) from the inducible *MET3* promoter on CEN plasmids was able to rescue the small cell size of *whi5Δ* mutants, with mean cell sizes of 42.1, 43.5, 43.2 and 42.4 fL, respectively (Fig. 3B). We note that the slightly larger cell size observed is likely due to constitutive expression of Whi5 from the *MET3* promoter, and was also observed by de Bruin et al [10]. These observations indicate that in wild type cells, phosphorylation of Whi5 on CDK sites or non-CDK sites is not essential for regulation of cell size. In addition, it has previously been shown that Whi5 overexpression in *cln3Δ* cells results in permanent cell cycle arrest in G1 prior to Start [9]. Similar to wild type Whi5, overexpression of Whi5-12Ala, Whi5-6Ala, or Whi5-18Ala phosphorylation mutants from the *GAL1* promoter on CEN plasmids caused lethality of *cln3Δ* cells (Fig. 3C), suggesting that hypo-phosphorylation of either CDK or non-CDK sites is not required for Whi5 to antagonize Cln3 activity.

### Specific CDK phosphorylation sites of Whi5 are required for function

Expression of various Whi5 phosphorylation mutants in wild type cells does not result in a dramatic phenotype [9], [10]. However, overexpression of Whi5-12Ala in cells that harbor the Swi6-SA4 mutant lacking four CDK sites results in a severe growth defect [9], suggesting that phosphorylation of either Whi5 or Swi6 is required for SBF activation and cell cycle promotion. To test if the non-CDK sites of Whi5 are similarly essential for SBF activation in the presence of Swi6-SA4, Whi5 mutants containing alanine substitutions for the 6 non-CDK sites (Whi5-6Ala), and all CDK and non-CDK sites (Whi5-18Ala) were tested in this assay. In cells expressing wild type Swi6, all of the *WHI5* constructs, *WHI5-WT*, *WHI5-12Ala*, *WHI5-6Ala*, or *WHI5-18Ala* expressed from the *GAL1* promoter on CEN plasmids, showed no change in growth rate as detected in this assay (data not shown). However, in combination with *SWI6-SA4*, *WHI5-12Ala* and *WHI5-18Ala* cells were inviable, whereas cells expressing *WHI5-WT* or *WHI5-6Ala* were viable (Fig. 3D). Thus, phosphorylation of Whi5 non-CDK sites is not necessary for SBF activation in a *SWI6* or *SWI6-SA4* genetic background. We are currently unable to ascribe a biological function for phosphorylation of Whi5 at the 6 non-CDK sites.

**Figure 4:**
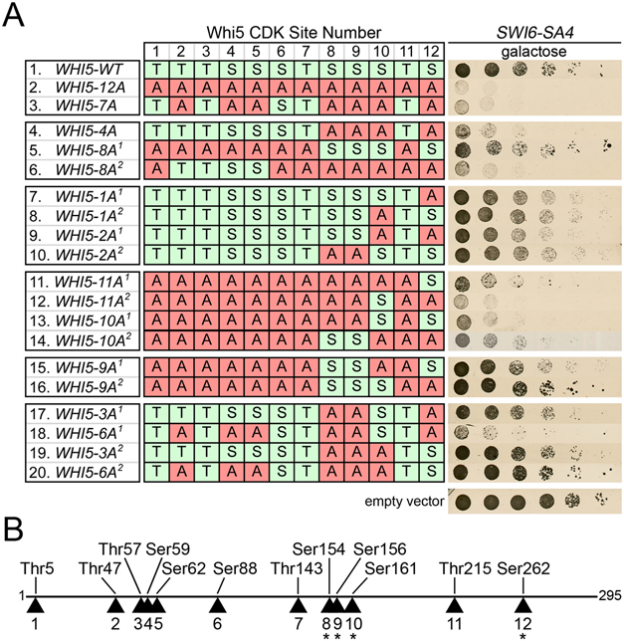
Mutational analysis of Whi5 phosphorylation sites. (A) *swi6Δ [SWI6-SA4]* cells expressing Whi5-WT or Whi5 phosphorylation mutants from the *GAL1* promoter. Cells were spotted in serial 5 fold dilutions on galactose media and incubated 48 hours. The table indicates the CDK sites of Whi5 that were left wild type (S/T) or mutated to alanine (A) for each mutant construct. (B) Schematic of the Whi5 protein showing relative location of CDK sites, numbered 1–12.

Although phosphorylation of multiple Whi5 CDK sites was detected *in vivo* (Fig. 1 and de Bruin et al.) [10], it is not known whether specific CDK sites or a threshold amount of phosphorylation determines Whi5 inactivation and dissociation from SBF. To test these two competing hypotheses of Whi5 inactivation, we performed a mutational analysis of Whi5 CDK sites and assayed for genetic interaction with the *SWI6-SA4* allele (Fig. 4). Consistent with previous reports [9], none of the Whi5 CDK mutants assayed caused growth defects when induced in cells expressing wild type Swi6 (data not shown). Similar to *WHI5-12Ala*, expression of *WHI5-7Ala* (CDK sites 2, 3, 5, 8, 9, 10, and 12 mutated to alanine) [10] caused lethality in combination with *SWI6-SA4* (Fig. 4, lines 2 & 3). Based on a preliminary survey of phosphorylation site mutants, we initially focused our attention on the C-terminal CDK phosphorylation sites. Four C-terminal CDK sites of Whi5 (8, 9, 10, and 12) as a group were necessary and sufficient for viability with Swi6-SA4. When *WHI5-4Ala*, with the four sites mutated to alanine, is expressed in combination with *SWI6-SA4*, cells were inviable (Fig. 4, line 4). In contrast, when *WHI5-8Ala^1^*, where the other 8 CDK sites in Whi5 are mutated to alanine, is expressed with *SWI6-SA4*, cells were viable (Fig. 4, line 5). This demonstrates that CDK sites 8, 9, 10, and 12 are specifically required for Whi5 inactivation in this assay. To rule out the possibility that phosphorylation of any four CDK sites was sufficient to inactivate Whi5, several constructs having four or more wild type CDK sites were also analyzed. For example, when four N-terminal CDK sites (2, 3, 4, and 5) are wild type while the other eight CDK sites are alanine in *WHI5-8Ala^2^* (Fig. 4, line 6), the *SWI6-SA4* cells were still inviable, suggesting that the four N-terminal sites were not sufficient to allow for viability.

Additional analysis of CDK sites 8, 9, 10, and 12 was performed to assess their function as single sites, pairs of sites, or in groups of three. Mutation of site 10 or 12 alone, or pairs of sites (10 and 12; or 8 and 9) to alanine, while leaving the other CDK sites wild type did not affect cell growth (Fig. 4, lines 7–10). This demonstrates that no single C-terminal CDK site or pair of sites is necessary for viability. Conversely, mutating all other CDK sites to alanine, leaving only single CDK sites 10 or 12, or pairs of sites (10 and 12; or 8 and 9) wild type did not permit cell growth (Fig. 4, lines 11–14). This indicates that phosphorylation of a single site or pair of sites is not sufficient to support cell viability. Interestingly, when three CDK sites (8, 9, and 12; or 8, 9, and 10) were left wild type, they were sufficient to allow growth (Fig. 4, line 15), indicating 3 out of the 4 C-terminal CDK sites are sufficient for viability. Because these mutants with only three functional CDK sites are viable, the specific C-terminal CDK sites are adequate to promote Whi5 inactivation. However, Whi5 regulation by phosphorylation is complex in that some combinations of C-terminal mutations with other CDK sites influenced viability (Fig. 4, lines 17 & 18, lines 19 & 20). While CDK sites 8, 9, 10, and 12 are both necessary and sufficient for viability in combination with Swi6-SA4, supporting a model of specific sites utilized for inactivation, other CDK sites are able to support inactivation in certain combinations.

### Localization of Whi5 CDK mutants

**Figure 5:**
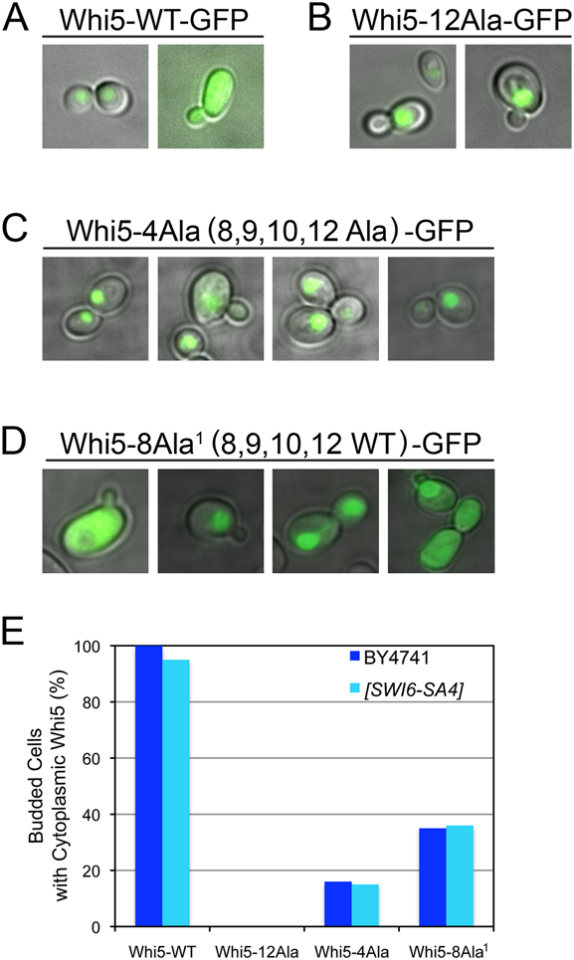
Cellular localization of Whi5 phosphorylation mutants. (A) Cultures containing *GAL1-WHI5-WT-GFP*, (B) *GAL1-WHI5-12Ala-GFP*, (C) *GAL1-WHI5-4Ala-GFP*, or (D) *GAL1-WHI5-8Ala-GFP* on a CEN plasmid were grown in galactose media and photographed for Whi5-GFP fluorescence in unbudded and budded cells. Representative cells are shown. (E) Graph of Whi5 localization in budded cells, showing the percentage of budded cells with cytoplasmic Whi5 in either wild type BY4741 or *swi6Δ [SWI6-SA4]* cells.

In wild type cells, Whi5 localization is nuclear in early G1, then exported to the cytoplasm just prior to budding [9]. Since Swi6-SA4 is constitutively localized in the nucleus [16], we hypothesized that Whi5 CDK mutants that caused lethality with Swi6-SA4 might also have defects in nuclear export. Localization of GFP tagged Whi5 was examined in wild type cells. As previously reported [9], Whi5-WT-GFP is nuclear in unbudded cells and cells just exiting mitosis, but is cytoplasmic in budded cells (Fig. 5A). However, Whi5-12Ala-GFP was localized to the nucleus in both unbudded and budded cells (Fig. 5B), confirming that CDK phosphorylation is required for export [9]. The localization of Whi5-4Ala-GFP, containing mutations of CDK sites 8, 9, 10, and 12, is nuclear in unbudded cells (Fig. 5C), but is cytoplasmic in only 16% of budded cells (Fig. 5C, 2^nd^ panel and Fig. 5E). Whi5-8Ala^1^-GFP, with CDK sites 8, 9, 10, and 12 wild type, is nuclear in unbudded cells, and is cytoplasmic 35% of budded cells (Fig. 5D and 5E). The localization of Whi5-WT-GFP and CDK mutants was also examined in *SWI6-SA4* cells, but was not different than localization observed in wild type cells (Fig. 5E). These observations suggest that in wild type or *SWI6-SA4* cells, Whi5 C-terminal CDK sites 8, 9, 10, and 12 influence localization, but are not entirely necessary or sufficient for wild type localization.

### *MSN5* is required for Whi5 nuclear export

**Figure 6:**
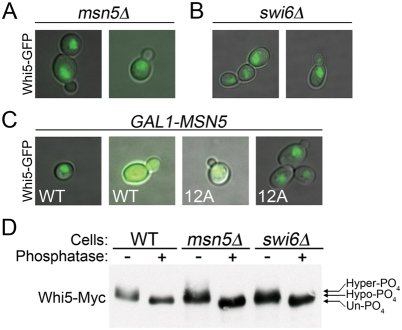
MSN5 is required for Whi5 nuclear export. (A) *msn5Δ* cells, or (B) *swi6Δ* cells containing *GAL1-WHI5-WT-GFP* CEN plasmid were grown in galactose media and photographed for Whi5-GFP fluorescence in unbudded and budded cells. Representative cells are shown. (C) Cells expressing *MSN5* from a *GAL1* promoter integrated at the endogenous locus contain either *GAL1-WHI5-WT-GFP* or *GAL1-WHI5-12Ala-GFP* CEN plasmids. Cells were grown and photographed as above. (D) Anti-Myc western blot of immunopurified Whi5-WT-13xMyc from wild type cells, *msn5Δ*, or *swi6Δ* cells untreated or treated with lambda phosphatase. Arrows indicate presence of three different migrating species.

Swi6 nuclear export is dependent on the karyopherin Msn5 [35]. Because Swi6 and Whi5 interact [9], [10], we examined whether Whi5 nuclear export was also Msn5 dependent. In *msn5Δ* cells, GFP tagged Whi5-WT was constitutively nuclear, even in budded cells (Fig. 6A). Whi5-WT was also nuclear in *swi6Δ* cells (Fig. 6B), suggesting that Swi6 is also involved in Whi5 nuclear export. Msn5 overexpression from the *GAL1* promoter was not sufficient to induce ectopic nuclear export of Whi5-WT, as its localization remains nuclear in unbudded cells (Fig. 6C). Additionally, *GAL1-MSN5* was unable to induce export of Whi5-12Ala and localization remained constitutively nuclear (Fig. 6C). Similarly, *GAL1-MSN5* was unable to rescue the growth defect of *GAL1-WHI5-12A SWI6-SA4* co-expressing cells (data not shown). Because Msn5 is known to export phosphoproteins [35]–[38], we examined phosphorylation of Whi5 in *msn5Δ* and *swi6Δ* cells (Fig. 6D). Immunoprecipitation and phosphatase treatment revealed that Whi5-WT was both hypo-phosphorylated and hyper-phosphorylated in *msn5Δ* and *swi6Δ* cells similar to wild type cells, indicating that the failure to export is not due to lack of Whi5 CDK phosphorylation.

### Whi5 C-terminal CDK sites affect cell size

**Figure 7:**
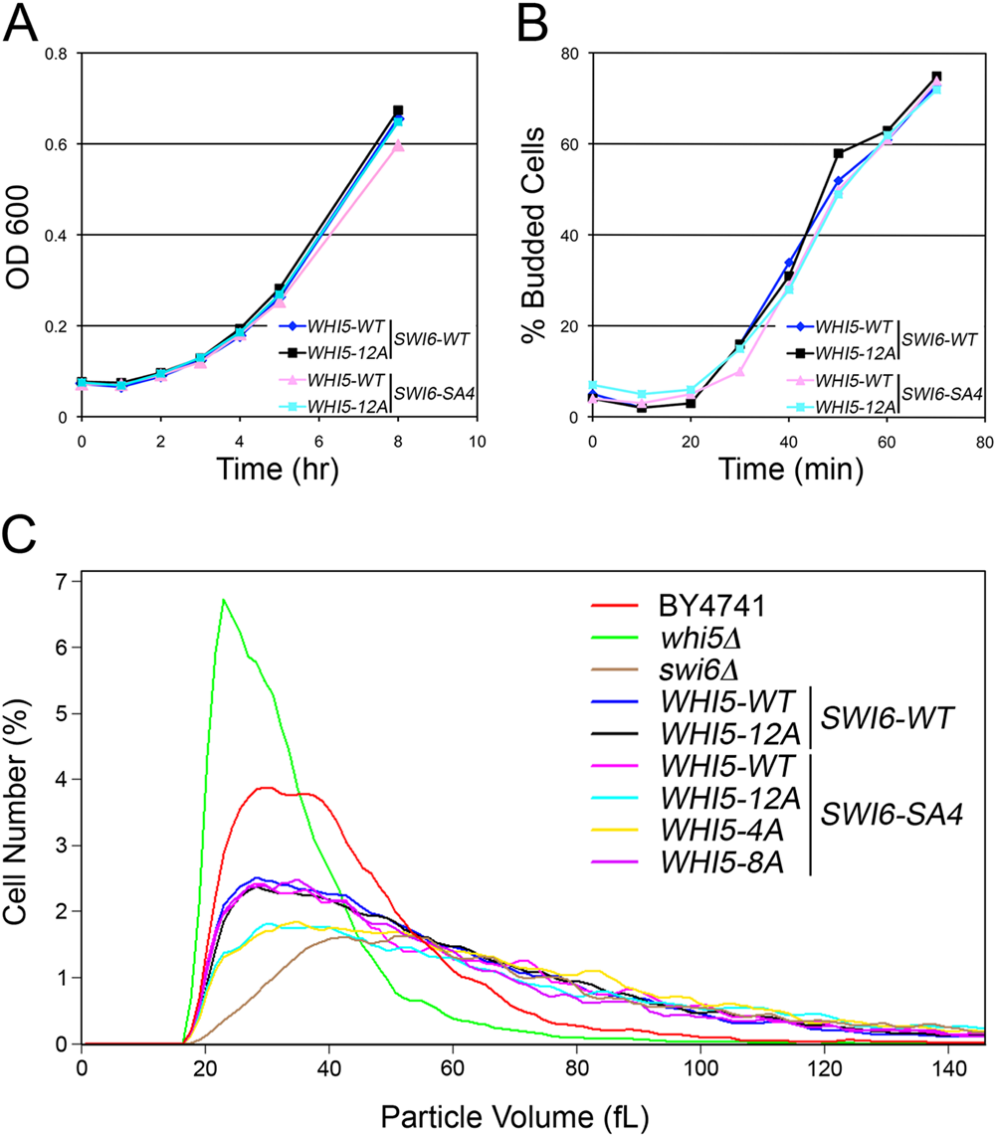
Whi5 C-terminal CDK sites are required for maintenance of cell size. (A) Growth rates measured by OD600 of *swi6Δwhi5Δ* cells expressing Swi6-WT or Swi6-SA4, and Whi5-WT or Whi5-12Ala from the 545 bp *WHI5* promoter fragment on CEN plasmids. (B) Budding index of the same strains as in A. Cells were synchronized with alpha factor and released into fresh media. Budded cells were counted every 10 minutes. (C) Cell size distribution analysis wild type cells (BY4741), *whi5Δ* cells, and *swi6Δ* cells compared to *swi6Δwhi5Δ* cells expressing Swi6-WT or Swi6-SA4, and Whi5-WT, Whi5-12Ala, Whi5-4Ala, or Whi5-8Ala from the 545 bp *WHI5* promoter fragment on CEN plasmids.

To determine if the four C-terminal CDK sites of Whi5 affect normal cell cycle progression, *WHI5-WT* and mutant alleles were expressed from the *WHI5* promoter in combination with *SWI6-SA4*. In contrast to cells overexpressing Whi5-12Ala from the *GAL1* promoter (as in Fig. 4), cells expressing Whi5-12Ala and Swi6-SA4 from their own promoters at physiologic levels are viable and grow at rates similar to cells expressing Whi5-WT and Swi6-WT (Fig. 7A) and have normal cell cycle profiles (data not shown). When cells were synchronized in G1 with mating pheromone and released, *WHI5-12A SWI6-SA4* cells budded at the same time and rate as *WHI5-WT SWI6-WT* cells (Fig. 7B). Although the cells displayed no overt growth defect, *WHI5-12Ala SWI6-SA4* co-expressing cells were large (Fig. 7C). The mean cell volume (fL) of cells co-expressing Whi5-12Ala and Swi6-SA4 was 67.6 fL, 40% larger than cells expressing wild type WHI5 and SWI6 with a mean of 48.2 fL, indicating that phosphorylation of either Swi6 or Whi5 is necessary for regulation of cell size. Cells with *WHI5-WT* and *SWI6-SA4*, or cells with *WHI5-12Ala SWI6-WT*, were similar in size distribution to *WHI5-WT SWI6-WT* cells, demonstrating that the effect on cell size is not observed in cells with either mutant alone. We also analyzed the cell size distribution of Whi5 CDK mutants. When expressed with Swi6-WT, Whi5-4Ala and Whi5-8Ala^1^ cells were similar in size to Whi5-WT and Whi5-12Ala (data not shown). In contrast, cells expressing Swi6-SA4 with Whi5-4Ala (CDK sites 8, 9, 10, 12 Ala) were larger in size, with a mean size of 62.0 fL, with a similar distribution as Swi6-SA4 Whi5-12Ala cells (Fig. 7C). Conversely, the size distribution of Swi6-SA4 cells with Whi5-8Ala^1^ (CDK sites 8, 9, 10, 12 WT), with a mean size of 54.6, was similar to that of cells containing both wild type alleles. These results demonstrate that in combination with Swi6-SA4, phosphorylation of Whi5 C-terminal CDK sites (8, 9, 10, and 12) is essential for maintenance of cell size.

## Discussion

### Whi5 CDK and non-CDK phosphorylation

Whi5 is a negative regulator of G1 cell cycle progression that is inactivated by CDK phosphorylation at Start [9], [10]. Whi5 contains 12 putative CDK phosphorylation sites. However, it has remained unclear if specific CDK phosphorylations sites on Whi5 are utilized for inactivation or if a critical phosphorylation threshold of randomly distributed CDK sites induces inactivation. Here we found that Whi5 is phosphorylated *in vivo* on 10 CDK sites and 6 novel, non-CDK sites. We also observed that Whi5 is found hypo-phosphorylated at all stages of the cell cycle, including early G1. We did not observe un-phosphorylated Whi5, even in mating pheromone arrested cells. Five out of 6 of the non-CDK sites are followed by acidic residues and conform to the consensus sequences for casein kinase I or casein kinase II (CKA1/2) [39], which is known to target transcription factors and RNA polymerases [40]. We also found that hypo-phosphorylation is not required for the repressive function Whi5 as *whi5Δ* cells expressing Whi5-18Ala (all CDK and non-CDK sites mutated to alanine) are normal in cell size and overexpression of Whi5-18Ala causes lethality in cln3Δ cells. Further experiments are needed to explore the biological function of Whi5 non-CDK phosphorylation and identify the kinase(s) responsible.

In addition to showing that Whi5 hypo-phosphorylation is not essential for inhibitory function, this analysis shows that phosphorylation is neither essential for Whi5 inactivation nor passage through Start. Whi5-12Ala expressed from the *WHI5* promoter fails to effect cell size distribution, indicating that the timing of cell cycle entry is not significantly affected by the phosphorylation site mutations. Our results differ from those of de Bruin et al, who report that Whi5-7Ala expressed from the *MET3* promoter caused a measurable defect in cell cycle progression, with cells inducing maximum SBF transcription at a ∼10% larger cell size [10]. The difference in results may be due to strain background differences or the use of the heterologous promoter. Nevertheless, the reported effect of Whi5-7Ala was not as significant as would be predicted if Whi5 phosphorylation was essential for SBF activation.

### Redundancy of Whi5 and Swi6 Phosphorylation

In contrast to the finding that Whi5 phosphorylation is not essential for cell size regulation at physiologic expression levels, phosphorylation of Whi5 and Swi6 becomes essential when Whi5 is overexpressed, as phosphomutants of these proteins synergize to cause lethality. Swi6 has 5 putative CDK phosphorylation sites; four of the five are in the N-terminal region, while the fifth CDK site is a part of the ankyrin repeat domain of Swi6. The *SWI6-SA4* mutant created by Sidorova, et al., that contains mutations of the four N-terminal CDK sites, does not affect periodic transcription of SBF targets (data not shown) [16]. While Swi6-SA4 alone does not have a significant cell cycle phenotype, Swi6-SA4 cells expressing *GAL1-WHI5-12Ala* are inviable and arrest as large, elongated, unbudded cells (data not shown) [9]. We exploited this phenotype in a mutational analysis of Whi5 CDK sites to identify four C-terminal CDK sites of Whi5, sites 8, 9, 10, and 12, that are necessary and sufficient to prevent lethality when Whi5 is overexpressed in combination with Swi6-SA4. Importantly, Whi5 retaining four or more N-terminal CDK sites intact was not sufficient to prevent lethality. This demonstrates that specific CDK sites of Whi5 are required for inactivation, rather than simply a threshold number. Consequently, we can exclude a model similar to that adopted for proteins such as Sic1 that require a threshold number of CDK phosphorylations to induce inactivation without a requirement for specific sites of phosphorylation [41]. These four phosphorylated residues of Whi5 may function to induce a conformational change or otherwise influence the interaction between Whi5 and SBF components. We conclude that phosphorylation of Whi5 and Swi6 is redundant, and that phosphorylation facilitates Whi5 inactivation.

CDK phosphorylation of Swi6 participates in the inactivation of Swi6 by inducing cytoplasmic localization [16], [18]. Swi6 Serine-160 is adjacent to a nuclear localization sequence and inhibits nuclear import once phosphorylated. When Ser-160 is mutated to alanine, as it is in the Swi6-SA4 mutant, this results in constitutive nuclear localization of Swi6 [16]. Whi5 is also exported from the nucleus upon CDK phosphorylation and a C-terminal phosphorylation mutant of Whi5 (sites 7–12 alanine) is largely nuclear [9]. Analysis of Whi5-GFP localization shows four C-terminal sites (8, 9, 10, and 12) are involved in nuclear export, as Whi5-4A was unable to be completely exported to the cytoplasm in budded cells, but are not sufficient to control localization, as Whi5-8Ala (8, 9, 10, and 12 wild type) remained nuclear in two thirds of budded cells. This suggests that the four CDK sites implicated in Whi5 inactivation differ from the CDK sites required to induce Whi5 nuclear export, and highlights a function for CDK sites 7 and 11 in Whi5 export. These data also indicate that Whi5 dissociation from SBF and its subsequent export to the cytoplasm are distinct steps requiring phosphorylation of different CDK sites.

Despite the dramatic cell cycle phenotype of *SWI6-SA4* cells when Whi5-12Ala is overexpressed, CDK phosphorylation of Whi5 and Swi6 is not essential for viability when Whi5 is expressed at physiologic levels. Cells expressing both Swi6-SA4 and Whi5-12Ala under control of their own promoters are completely viable, grow at normal rates, and enter the cell cycle after mating pheromone induced G1 arrest with normal kinetics. Their only apparent defect is a large cell size, suggesting that cells are delayed in passing Start when Whi5 and Swi6 cannot be phosphorylated. The four Whi5 CDK sites (8, 9, 10, and 12) identified in the overexpression assay with Swi6-SA4 are also necessary and sufficient to promote cell size regulation, as *WHI5-4Ala SWI6-SA4* cells were equivalent in size to *WHI5-12Ala SWI6-SA4* cells. Conversely, *WHI5-8Ala^1^* (CDK sites 8, 9, 10, and 12 wild type) cells were similar in size to *WHI5-WT* when combined with *SWI6-SA4*. The effect of these mutants on cell size indicates that the four specific CDK sites are important for the timing of Whi5 inactivation, even at physiologic expression levels. However, these data also imply that Whi5 and Swi6 are not essential targets of Cln-Cdc28 for cell viability when expressed at physiologic levels, and phosphorylation of Swi6 or the four sites of Whi5 is only required for proper coordination of cell size with cell cycle entry. In the absence of available CDK sites on Whi5 or Swi6, cells must use a mechanism other than CDK phosphorylation to activate SBF transcription. It is possible that the cell cycle activator Bck2 plays a role under these conditions, given its ability to activate SBF transcription independently of Cdc28 activity [42], [43].

### Parallels between Whi5 and Rb

The pathway of transcriptional activation in yeast is functionally similar to the mechanism of cell cycle entry in mammalian cells. Whi5 is a functional analog of the Retinoblastoma tumor suppressor protein that also represses transcription in early G1. Previous studies from our laboratory show that Rb bound to E2F transcription factors in early G1 is hypo-phosphorylated on CDK phosphorylation sites [24]. Whi5 is similarly hypo-phosphorylated in early G1, but hypo-phosphorylation is not necessary for its repressive function. Further experiments are needed to determine the requirement for Rb hypo-phosphorylation in repression of E2F promoters. Additionally, phosphopeptide maps of hyper-phosphorylated Rb during late G1 are nearly identical to hypo-phosphorylated Rb found in early G1 [44], [45], indicating that individual CDK sites are targeted equally in Rb phosphorylation and suggesting specific sites do not have a significant function. This suggests a model of Rb regulation where inactivation by hyper-phosphorylation is achieved by phosphorylation of a threshold number of CDK sites [28]. In contrast to that model for Rb, we identified specific CDK sites that function in Whi5 inactivation and regulation of cell size. Because the regulatory pathways of cell cycle entry are largely conserved between yeast and mammalian cells, the mechanism of regulation by specific phosphorylation observed for Whi5 suggests further investigation of Rb phosphorylation in mammalian systems is warranted.
